# Evaluating the ability of community‐protected forests in Cambodia to prevent deforestation and degradation using temporal remote sensing data

**DOI:** 10.1002/ece3.4492

**Published:** 2018-10-01

**Authors:** Minerva Singh, Damian Evans, Jean‐Baptiste Chevance, Boun Suy Tan, Nicholas Wiggins, Leaksmy Kong, Sakada Sakhoeun

**Affiliations:** ^1^ Imperial College London South Kensington London UK; ^2^ École française d'Extrême‐Orient Paris France; ^3^ Phnom Kulen Program, Archaeology and Development Foundation London UK; ^4^ Angkor International Research and Documentation Centre APSARA National Authority Siem Reap City Siem Reap Province Cambodia; ^5^ School of Earth and Environmental Sciences The University of Queensland St Lucia QLD Australia

**Keywords:** AGB stock, ALOS PALSAR, LiDAR, protected areas

## Abstract

Community forests are known to play an important role in preserving forests in Cambodia, a country that has seen rapid deforestation in recent decades. The detailed evaluation of the ability of community‐protected forests to retain forest cover and prevent degradation in Cambodia will help to guide future conservation management. In this study, a combination of remotely sensing data was used to compare the temporal variation in forest structure for six different community forests located in the Phnom Kulen National Park (PKNP) in Cambodia and to assess how these dynamics vary between community‐protected forests and a wider study area. Medium‐resolution Landsat, ALOS PALSAR data, and high‐resolution LiDAR data were used to study the spatial distribution of forest degradation patterns and their impacts on above‐ground biomass (AGB) changes. Analysis of the remotely sensing data acquired at different spatial resolutions revealed that between 2012 and 2015, the community forests had higher forest cover persistence and lower rates of forest cover loss compared to the entire study area. Furthermore, they faced lower encroachment from cashew plantations compared to the wider landscape. Four of the six community forests showed a recovery in canopy gap fractions and subsequently, an increase in the AGB stock. The levels of degradation decreased in forests that had an increase in AGB values. However, all community forests experienced an increase in understory damage as a result of selective tree removal, and the community forests with the sharpest increase in understory damage experienced AGB losses. This is the first time multitemporal high‐resolution LiDAR data have been used to analyze the impact of human‐induced forest degradation on forest structure and AGB. The findings of this work indicate that while community‐protected forests can improve conservation outcomes to some extent, more interventions are needed to curb the illegal selective logging of valuable timber trees.

## 1 INTRODUCTION

Protected areas (PAs) are widely regarded as an important bulwark against deforestation and biodiversity loss (Klein et al., 2015). However, PAs are a partial and imperfect conservation solution for the tropical forests of the world. Analysis of the Global Forest Cover Change dataset (Hansen et al., 2013) revealed that globally, protected areas lost 3% of their forest cover, intact forest landscapes have lost 2.5%, while protected intact forest landscapes have lost 1.5% of their forest cover (Heino et al., 2015). An analysis of 60 tropical forest protected areas revealed that half of these have faced significant biodiversity erosion as a result of forest resource extraction and wildlife hunting. Furthermore, overharvesting, deforestation, and degradation outside the reserve boundaries can have a detrimental effect on biodiversity persistence within the reserve boundaries (Laurance et al., 2012).

International Union for Conservation of Nature (IUCN) categorizes protected areas into six broad management categories, ranging from strict nature reserves that limit human activity to reserves that allow for sustainable resource extraction (Hayes et al., 2013). Categories V and VI are more amenable to human resource extraction compared to categories I and II which focus more on preserving natural features and curtailing human activities within the boundaries of the PA (IUCN, 2013). In addition to enforcement, PA efficacy also depends on the benefits and compensation accrued by local communities (Liu et al., 2001). Strict PA categorizations which do not provide benefits to the local community can also cause forest loss to worsen. A temporal analysis of land‐cover change in the Wolong PA, an IUCN Category I PA in China, revealed that the area had undergone considerable degradation subsequent to its designation as a PA (Liu et al., 2001). On the other hand, cooperation of the local communities can increase the chances of PAs being able to avoid forest loss. A Latin America‐wide study carried out by Porter‐Bolland et al. (2012), which compared annual forest loss within 40 protected areas and 33 community‐managed forests, discovered that the latter had a lower rate of annual forest loss. The authors suggest that accounting for local tenure rights and the socio‐economic welfare of the local inhabitants can yield better conservation outcomes. Community forestry was found to better protect forests from anthropogenic disturbances and logging in the Prey Long district of Cambodia where a substantial proportion of people depend on forests for sustenance (Lambrick, Brown, Lawrence, & Bebber, 2014). However, satellite land cover change analysis from 2003 to 2013 indicated that protected areas in Paraguay's Atlantic forest helped slow deforestation (Da Ponte et al., 2017a). A meta‐analysis of African and Latin‐American PAs revealed that while strict PAs delivered fire‐prevention benefits, multiuse community PAs were more effective in fire prevention and could contribute to both biodiversity conservation and AGB stock retention (Nelson & Chomitz, 2011). Community forests can facilitate long‐term forest protection in certain situations and deliver benefits to the local community (Bray et al., 2008). On the basis of the existing literature, it may be inferred that both community forests and protected areas deliver different outcomes across different regions. Evaluating the ability of different protection schemes, to counter forest cover change (in the form of deforestation and degradation) is important (Da Ponte, Roch, Leinenkugel, Dech, & Kuenzer, 2017b).

Different magnitudes of forest degradation and regeneration impact forest structure parameters such as AGB storage and canopy structure‐related variables such as gap fraction differently. In fact, even low logging volumes can lead to a decline in the carbon stocks of tropical forests (Bryan, Shearman, Ash, & Kirkpatrick, 2010). However, forests regenerating after shifting cultivation are vital AGB sinks and their ability to store biomass increases with the length of abandonment (Mukul, Herbohn, & Firn, 2016). Forest regeneration and associated increases in forest cover facilitate a rapid increase in carbon stocks storage (Lohbeck, 2016). Even degraded forests can act as valuable carbon sinks under certain conditions (Alamgir et al., 2016).

In addition to the AGB storage, other forest structure parameters such as canopy cover and tree height also vary across a degradation gradient (Mehta, Sullivan, Walter, Krishnaswamy, & DeGloria, 2008; Pfeifer et al., 2016). Gap fractions in the forest canopy (open gaps in forest canopy not covered by foliage) vary considerably between primary forests compared and forests that have been logged (Pinagé, Matricardi, Osako, & Gomes, 2014). In the Brazilian Amazon, it was discovered that forest canopy gaps undergo rapid regeneration and that within a three‐year period, and no detectable difference remained between the canopy gaps of undisturbed and logged forests (Espirito‐Santo, Keller, Braswell, & Palace, 2006). Canopy gaps resulting from conventional logging had lower rates of recovery compared to those caused by reduced‐impact‐logging 3.5 years after logging in the Brazilian Amazon (Asner, Keller, Pereira, Zweede, & Silva, 2004).

Different types of remote sensing (RS) data have been used (either alone or in conjunction with each other) to study the variation in forest structure, greenness and degradation in time and space for tropical forests. Optical data such as those derived from Landsat have been extensively used for mapping temporal changes in forest cover and land use types in the tropics (Potapov et al., 2014). A freely available software system CLASlite developed by the Carnegie Institute of Science has employed Landsat data to detect temporal forest cover change in Madagascar (Allnutt, Asner, Golden, & Powell, 2013) and distinguish plantations from natural forests in Borneo (Bryan et al., 2013). In addition to Landsat data, ALOS PALSAR radar data have been employed for studying the patterns of forest degradation and recovery in Cambodia, Laos, and Vietnam (Mermoz & Le Toan, 2016) and mapping varying levels of forest degradation in Laos (Singh, Tokola, Hou, & Notarnicola, 2017). LiDAR data, which have a higher spatial resolution than optical and radar data, have also been extensively used for mapping the variation in AGB stocks and other forest structure parameters in degraded forests of tropical Asia (Kronseder, Ballhorn, Böhm, & Siegert, 2012; Singh et al., 2016). The ability of LiDAR to capture canopy height at a fine scale makes it a useful tropical forest mapping tool with other datasets, notably Landsat (Leinenkugel, Wolters, Oppelt, & Kuenzer, 2015; Peou, Natarajan, Tianhua, & Philippe, 2016). LiDAR data offer the distinct advantage of being able to identify individual trees and measures of their biophysical parameters such as height, crown volume, and area. These measurements in turn can be used to model AGB variation at a landscape scale (DeFries, Rudel, Uriarte, & Hansen, 2010; Motzke, Wanger, Zanre, Tscharntke, & Barkmann, 2012). Temporal LiDAR data have been used for monitoring the impact of selective logging on AGB stocks in the Brazilian Amazon. Using these data, locations that had lost their tallest trees were identified, along with changes in the proportion of logging trails, landings, and gaps. Furthermore, the role of large tree removal in influencing AGB stocks from 2010 to 2011 was examined (Asner, Knapp, Balaji, & Páez‐Acosta, 2009).

RS data can play a vital role in monitoring the efficacy of conservation management schemes and PAs. Landsat data have been extensively used for examining the ability of protected areas to retain forest cover at both local and global scales (Allnutt et al., 2013; Heino et al., 2015). While ALOS‐PALSAR and airborne LiDAR data have not been extensively used for mapping and monitoring the efficacy of protected areas and conservation management schemes, we hypothesize that use of these different RS data can help to monitor different aspects of tropical forest cover change dynamics (their impacts on forest structure dynamics such as AGB) and improve our understanding of the ability of community‐protected forests to retain forest cover and prevent degradation in Cambodia.

The main objective of this research was to compare the temporal variation in forest cover (including the expansion of cashew plantations) and structure of six different community forests located in a National Park in Cambodia and assess how these dynamics vary between this community‐protected forests and the wider study area.

The specific aims of this study are as follows:

1. to quantify the variation in Landsat‐derived forested areas in the study area and across the different community‐protected forests;
2. to use a combination of LiDAR and Landsat data to map and monitor the changes in forest cover, cashew plantation, and bare soil from 2012 to 2015;
3. to quantify the variation in LiDAR‐derived forest structure parameters (such as AGB) from 2012 to 2015; and
4. to quantify the variation in the vegetation greenness and forest degradation for both the study area and the different community forests.

In addition to these, the impact of selective logging from 2012 to 2015 will be estimated by identifying tree height classes that have faced losses (at the individual tree scale).

It is expected that the findings of our research will help us to quantify the efficacy of community‐protected forests in preventing deforestation and facilitating regeneration (compared to the wider landscape) and their ability to curb the selective illegal logging of individual tree species. The countries of Greater Mekong region have high rates of deforestation resulting from factors ranging from plantation agriculture to selective logging, with the latter being more difficult to detect (Leinenkugel et al., 2015). Quantifying the ability of community forests to facilitate forest regeneration and curb selective logging can help to inform conservation management strategies.

## 2 MATERIALS AND METHODS

### 2.1 Study area

**Figure 1:**
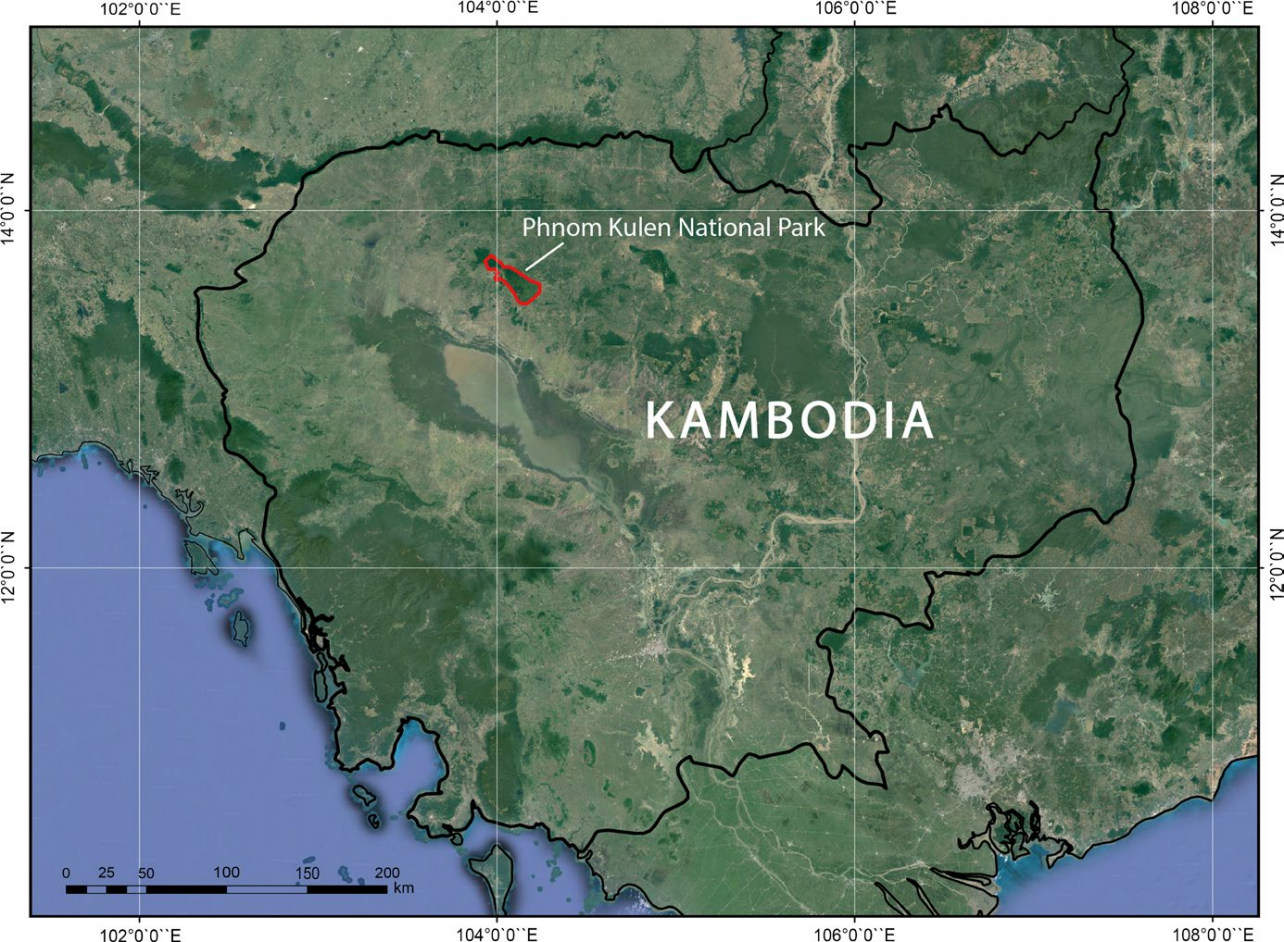
Phnom Kulen National Park (PKNP), Cambodia

Phnom Kulen National Park (PKNP) is located 48km from Siem Reap in northwestern Cambodia (Figure 1). It is an important archaeological site, a critical area for biodiversity, and a significant component of the regional watershed which includes the World Heritage listed Angkor Archaeological Park. In terms of composition, PKNP is mainly dominated by semi‐evergreen forests (with isolated patches of dry dipterocarp forests). However, in terms of land use forests that have undergone varying levels of degradation as a result of activities such as selective logging and swidden agriculture and land use types such as cashew plantations now dominate PKNP. Notably, PKNP is home to several IUCN‐listed species of international concern, including the Pileated Gibbon, Indochinese Silver Langur, Bengal Slow Loris, and Binturong (Hayes et al., 2013; Peou et al., 2016).

Over recent decades, Cambodia has experienced some of the highest rates of deforestation globally (Hansen et al., 2013). Despite its protected status, PKNP has experienced high deforestation and degradation rates and, as with other PAs in Cambodia, faces significant threats from local resource extraction activities (Motzke et al., 2012). Additionally, several villages are located within or on the boundary of PKNP. Many of these villages have experienced significant population growth due to people relocating in search of cheap farmland (DeFries et al., 2010). Furthermore, the villages located within the boundary of PKNP have high rates of poverty, low educational levels, and depend heavily on forest resources for their sustenance.

**Figure 2:**
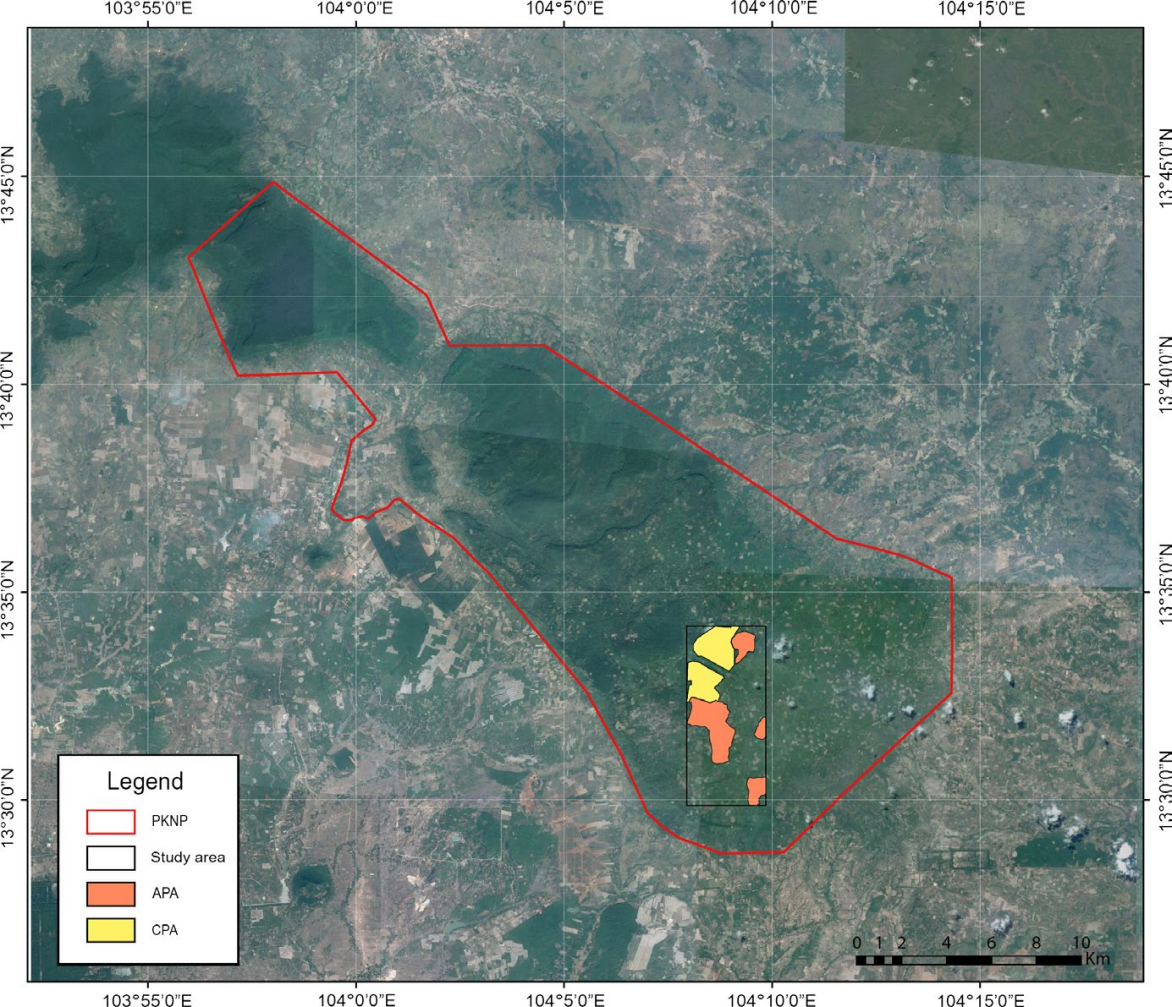
Location of study area and community forests in PKNP

Community‐protected areas (CPAs) were established in different parts of PKNP 2001 onwards with assistance from the Food and Agriculture Organization (FAO). The purpose of these CPAs was to promote the participation of local communities in forest conservation and allow the recovery of forest resources. The study area under consideration is a 32 km^2^ mixed forest‐cashew plantation landscape located in the southwest portion of PKNP (Figure 2). It comprises of two CPAs: Anlong Thom (CPA‐AT: 2.7 km^2^) and Khlah Khmum (CPA‐KK: 3.06 km^2^). Aside from the two CPAs, Archaeological‐Protected Areas (APAs) have also been created within PKNP to protect vulnerable archaeological sites and forests from destructive agricultural practices and to build awareness in the local population about the natural environment and historical heritage (LiForest, 2016). In addition to the CPAs, four APAs are present in our study area: Neak Ta (APA‐NT: 0.52 km^2^), Khlah Khmum (APA‐KK: 0.94 km^2^), Rong Chen (APA‐RC: 3.98 km^2^), and Thma Dap (APA‐TD: 1.48 km^2^). While the establishment of the CPAs and APAs was driven by slightly different motives, the management plans of both the community forest types seek to prevent forest loss within their bounds and reduce human encroachment.

### 2.2 Field data collection

Field survey was conducted in March 2016 and during this the geo‐locations of the different land cover types, including, forests, cashew plantations and bare earth were collected. The standard FAO definitions of forests (which is common for all the countries in the world) was used; “land of at least 0.5 ha covered by trees higher than 5 m and with a canopy cover of more than 10%, or by trees able to reach these thresholds, and predominantly under forest land use” (Hansen et al., 2013). The survey revealed that the community forests were comprised mostly of regenerating/secondary forests while areas outside these were comprised of severely degraded forests, agricultural/bare areas and cashew plantations. Bearing in mind the criticism of the bespoke standard definition and for the purpose of this research, cashew plantation monocultures were given their own category as opposed to categorizing them as forests. The field‐collected geo‐locations were cross‐verified using high‐resolution Google Earth imagery.

### 2.3 Remote sensing data used

#### 2.3.1 Spaceborne optical and radar data

Spaceborne optical and radar data in the form of Landsat TM and ALOS PALSAR, respectively, were used in this research. Landsat TM data (path 127, row 51 with spatial resolution 30m) for March 2011–2015 were downloaded from Earth Explorer. The month of Landsat data acquisition was selected to match the season of LiDAR data acquisition. Raw Landsat data were converted to surface reflectance by applying both radiometric and atmospheric correction to these data through the freely available software CLASlite (Asner, Lactayo, Tupayachi, & Luna, 2013; Asner et al., 2009). In addition to atmospheric corrections, masking of clouds and haze was carried out by the software. An Automated Monte Carlo Un‐mixing algorithm that uses a probabilistic subpixel analysis approach was used to decompose the surface reflectance data into fractional cover (Asner et al., 2009). Under the subpixel analysis approach, each pixel of the data is decomposed into a fraction of photosynthetic vegetation (PV), nonphotosynthetic vegetation (NPV), and bare substrate (BS). The recommended threshold values of PV > 80% and BS < 20%were used to decompose the fractional cover image into binary maps of forest and nonforest cover (Asner et al., 2009; Bryan et al., 2013). The binary maps of forest–nonforest areas have been extensively derived using CLASLite for other parts of the world including the Peruvian Amazon where they helped to identify the increased forest cover loss within the protected areas as a result of mining (Asner & Tupayachi, 2017).

The surface reflectance data obtained from CLASlite were used to derive the Normalized Vegetation Index (NDVI), which is a commonly used indicator of vegetation greenness and health. More degraded ecosystems have lower values of NDVI (Meneses‐Tovar, 2011). NDVI values for both 2011 and 2015 were computed from the near infra‐red and red bands of the Landsat data. ALOS PALSAR data with a spatial resolution of 25m were downloaded from the JAXA website (EORC‐JAXA, 2016). These data are available at 5‐year intervals and were thus downloaded for 2010 and 2015. These data have dual polarization: HH (horizontal transmit, horizontal receive) and HV (horizontal transmit, vertical receive) and are provided in the Digital Number (DN) format. In order to obtain the backscatter values, the DN values of both HH and HV were converted to the normalized radar cross‐section (σ^0^) (Avtar, Suzuki, Takeuchi, & Sawada, 2013). An Enhanced Lee filter was applied to reduce speckles. HH and HV values were used to calculate the Radar Forest Degradation Index (RFDI), a radar‐derived measure of forest degradation (Mitchard et al., 2011) using the Equation (1) (Singh et al., 2017):

(1) RFDI=(HH−HV)/(HH+HV)

The backscatter values of HH and HV are strongly associated with the forest structural components, orientation, and canopy cover (Mitchard et al., 2011). RFDI values are obtained on a scale of 0 to 1. When the canopy opens up (as a consequence of logging and deforestation), RFDI values go up. Completely cleared areas have RFDI value of 1, while undisturbed forests have RFDI values ranging from 0.3 to 0.4 (Saatchi, Houghton, Dos Santos Alvala, Soares, & Yu, 2007). Lower values of RFDI indicate higher levels of forest canopy cover and intactness (Singh et al., 2017).

#### 2.3.2 Airborne LiDAR

LiDAR data were acquired over the study area in March 2012 and April 2015 (Evans, 2016; Evans et al., 2013). For both data acquisitions, a Leica ALS60 laser system and a 40 megapixel Leica RCD105 medium‐format camera within an external pod were used by mounting to the left skid of a Eurocopter AS350 B2 helicopter (Evans, 2016). For our study area, the 2012 data were derived from discretized full waveform data acquired in both N‐S and E‐W strips, while the 2015 data represent a combination of discrete‐return data (NW‐SE strips) and discretized points from full waveform data acquired in NW‐SE strips. The point density of the 2012 LiDAR data was 12 points/m^2^ (Singh et al., 2016) and the LiDAR data collected in 2015 were>15 points/m^2^ (Evans, 2016). In order to achieve this level of accuracy and point density, was achieved by flying at altitudes of 800–1000 m above‐ground level at a speed of 80 knots, with the ALS70 configured to Multipulse in Air (MPiA). The pulse rate was 500 kHz with a scan angle of 45° from nadir and a swath side‐lap of 50% (i.e., almost all terrain was scanned twice from different angles). Aircraft attitude was measured by a Honeywell CUS6 IMU at a rate of 200 kHz and positional data was logged at 2 Hz using a survey‐grade L1/L2 GNSS receiver mounted in the tail rotor assembly (Evans, 2016). These data were classified into ground and nonground points using the LiForest software (LiForest, 2016). It has been suggested that LiDAR data with point density >0.5 pulses/m^2^ produce reliable estimates of the forest canopy (Hansen et al., 2013) and LiDAR data with pulse density greater than 1 pulse/m^2^ have limited impact on estimating forest structure variables (Andersen, Reutebuch, McGaughey, d'Oliveira, & Keller, 2014). Hence, no thinning of the 2015 LiDAR dataset was carried out.

Ground returns were used to derive a Digital Elevation Model (DEM) while vegetation returns were used to generate a Canopy Height Model (CHM) giving the upper boundary of the canopy (Popescu & Zhao, 2008). The CHM and DEM were generated at a resolution of 1m (LiForest, 2016). Furthermore, LiForest software was used for isolating individual trees from the LiDAR data and extracting their individual locations, associated tree heights and crown area (LiForest, 2016). The individual scale LiDAR‐derived tree heights were scaled to the plot scale for both 2012 and 2015 (Singh et al., 2016).

Points with elevation values greater than the height break of 2m were considered to be tree points and the ratio of LiDAR returns less than the height break to the total number of returns was computed to produce estimates of gap fractions at a 15m resolution (LiForest, 2016). The resulting point densities for the 2012 data within our study area are 12 points/m^2^ for the 2015 data, point densities are 16 points/m^2^ (Evans, 2016). The gap fraction values range from 0% to 100% where 0 means a closed canopy and 100% means an open canopy [19]. Canopy cover was also extracted for the entire study area (Chen et al., 2014). Previous research conducted in the degraded tropical forests of Angkor Thom (which too are located in this region and have a similar land use context of forests; Singh et al., 2016) established a log–log‐based aerial data‐derived canopy cover allometric model is most robust for scaling up the field estimates of AGB and produces the best estimates of landscape scale forest biomass for the region (equation (2)).

(2) Ln(AGB)=6.05+2.828∗Ln(Canopy Cover)+error

This equation was used to produce AGB estimates for both 2012 and 2015 using the aerial imagery‐derived canopy cover as previously done in (Singh, Evans, Friess, Tan, & Nin, 2015; Singh et al., 2016). Similar log–log‐based models have been used for landscape scale biomass mapping with LiDAR‐derived variables in other tropical forest ecosystems as well (Réjou‐Méchain et al., 2015).

In addition to these, relative density models (RDMs) were computed from the LiDAR data of 2012 and 2015. This metric helps to map the impact of logging roads, skid trails, and landings. It is a raster layer indicating the percentage of LiDAR returns within a user‐specified above‐ground height category (Andersen et al., 2014). This was derived using LAStools by using the return data both 1m above the ground and from 1m to 10m above the ground as done previously (LiForest, 2016). High RDM values suggest a relatively intact understory with low skidding impact and lower RDM values indicate understory damage (Ellis, Griscom, Walker, Gonçalves, & Cormier, 2016).

### 2.4 Data analysis

Wilcoxon paired sample tests were applied to examine whether the LiDAR‐derived forest structure variables had changed significantly between 2012 and 2015. This is a nonparametric test that does not need the assumption of normally distributed data (Gaveau et al., 2009; Grandin, 2011). The temporal changes in LiDAR‐derived tree heights, crown area, and LiDAR‐derived % gap fraction from 2012 to 2015 were evaluated using Wilcoxon paired sample tests. Additionally, the temporal variation in NDVI from 2011 to 2015 and RFDI from 2010 to 2015 were examined using this technique as well. The temporal NDVI evaluates how the greenness value had changed between the different community forest areas in the given two time periods while RFDI evaluates the change in forest degradation for the two time periods. While the initial LiDAR data were collected in 2012, the initial Landsat and ALOS‐PALSAR data were collected in 2011 and 2010 respectively. In case of Landsat, the data were collected in March 2011 (same season as LiDAR 2012 acquisition) to minimize the phonological interference from seasonality. Further since very little large‐scale forest cover changes are reported to have occurred in the immediate study area after 2010, the 2011 Landsat and 2010 ALOS are deemed appropriate for our purpose.

CLASlite‐derived Landsat forest/nonforest maps were used to compute forest gain, loss and persistence from 2012 to 2015 using a cross‐tabulation module provided within the IDRISI Land Change Modeller (Kitzberger, Raffaele, & Veblen, 2005; Schulz, Cayuela, Echeverria, Salas, & Benayas, 2010). This is a statistical technique to identify patterns of land cover change and map spatial distributions of forest persistence, gain, and loss (Schulz et al., 2010). One of the leading causes of deforestation in the study area is the establishment of cashew plantation monocultures. The binary forest/nonforest cover maps derived from CLASLite cannot identify cashew plantations. Hence, a combination of Landsat‐derived NDVI and LiDAR‐derived canopy heights was used in conjunction with field data (locations of the different land use types) to produce a 3 class forest map (forests, cashew plantations, and bare earth) using a machine learning algorithm, random forests (RF) for both 2012 and 2015. RF is a nonparametric technique developed by Brieman in 2001 (Breiman, 2001). It is an ensemble‐based modelling technique where individual classifiers are built and later combined to improve predictive performance (Devaney, Barrett, Barrett, Redmond, & John, 2015). This technique can work with high‐dimensional data and correlated predictors and has thus been extensively used for land use classification (Gislason, Benediktsson, & Sveinsson, 2006). For classification purposes, an ensemble of individual decision‐tree classifiers is created and these are combined using a majority voting scheme. The individual trees are constructed using a bootstrap sample of the training data, whereby the training is performed on two‐thirds of the data samples and the remaining one‐third of the data samples are omitted. The latter is used for testing the robustness of the developed model (Devaney et al., 2015). In this research, the RF classification was carried out by using 70% of the data for training and 30% for testing in the R programming language.

## 3 RESULTS

### 3.1 Overall forest cover change patterns

**Figure 3:**
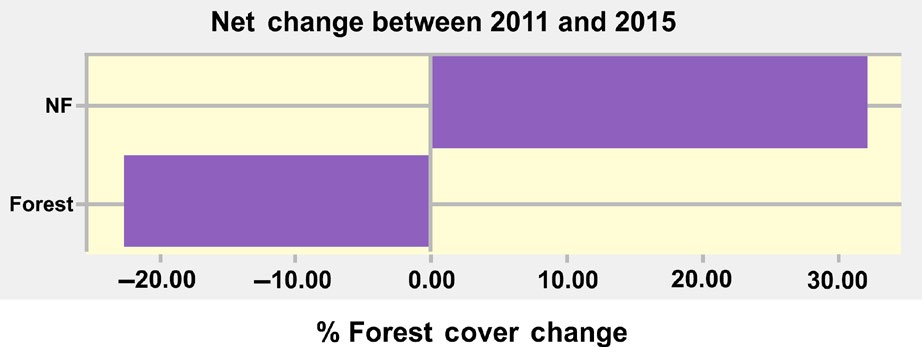
Landsat‐derived forest cover change from 2011 to 2015

The binary Landsat forest/nonforest cover maps derived for 2011 and 2015 were examined for the temporal changes in forest cover using IDRISI (Figure 3). It was discovered that over a 4‐year period, the forest cover in the study area had declined by more than 20%.

**Figure 4:**
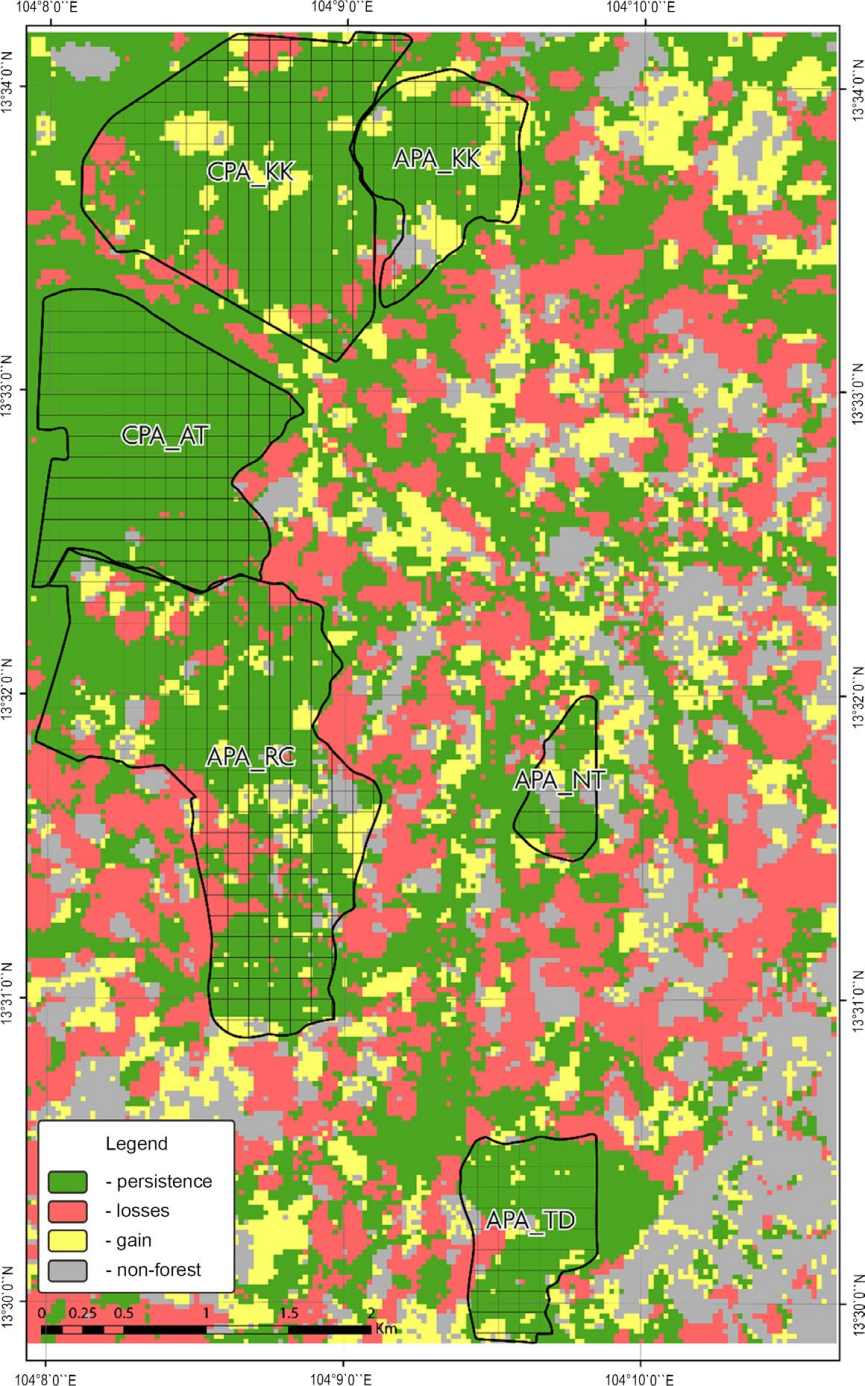
Forest persistence, gains, and losses from 2011 to 2015

The forest cover persistence and gain in the entire study area from 2011 to 2015 was 53% and 16%, respectively (Figure 4).

Compared to the overall study area, the persistence of forest cover was much higher in the community forests (Table 1).

**Table 1:** Forest cover persistence, gain, and loss in the community forests 2011–2015

	%Forest cover persistence	%Forest cover loss	%Forest cover gain
APA‐KK	71.9	3.6	24.6
APA‐NT	76.9	9.12	14
APA‐RC	72.4	14.3	13
APA‐TD	84.2	7.06	8.7
CPA‐KK	78	11	11.1
CPA‐AT	98.9	0.8	0.2

A visual inspection of the forest cover loss, gain, and persistence map (Figure 3) also reveals that the community‐protected forests are dominated by persistent forest cover.

Furthermore, the three class Landsat‐LiDAR forest maps developed for both the time periods using random forests showed high levels of accuracy. The 2012 Landsat‐LiDAR based 3 class forest cover map was found to have an overall accuracy of 98% and kappa of 0.97 on the test data. The 2015 Landsat‐LiDAR‐based 3 class forest cover map was found to have an overall accuracy of 99% and kappa of 0.98 on the test data.

Analysis of these two random forest‐based maps also indicated high levels of forest cover retention within the community forests. Further analysis of these data also revealed that overall forest cover declined by 20.4% across the entire study area. This is consistent with the findings of Figure 3, which also indicate a similar decline in forest cover values (with the Landsat‐based forest–nonforest maps).

**Figure 5:**
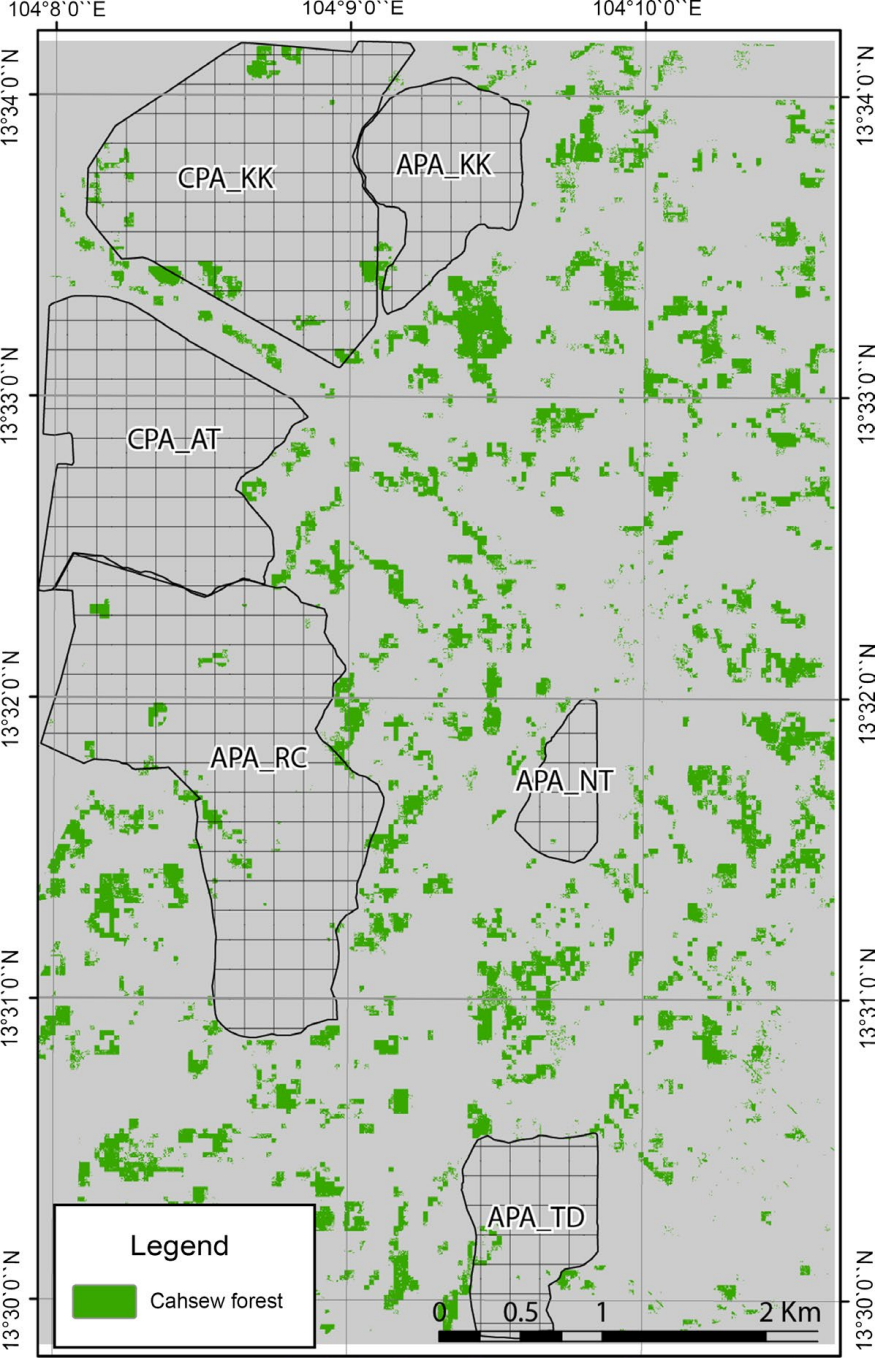
The spread of cashew plantations from 2011 to 2015

Analysis of the random forest‐derived Landsat‐LiDAR maps also revealed that cashew plantations increased by 7.5% and the bare ground increased by 70.8%. In all, 4 km^2^ of forests in the area were converted to cashew plantations from 2012 to 2015 (Figure 5). Figure 5 shows the areas converted to cashew plantations by 2015.

A visual examination of Figure 5 indicates that cashew plantations have penetrated the community forests marginally and that most of the forest‐cashew plantation conversions have occurred outside the community forests. In addition to examining the changes in forest cover and cashew plantation expansion, we have examined the spatial distribution of canopy height changes using LiDAR. Furthermore, the changes in forest structural and spectral properties within the community forests from 2012 to 2015 were also examined.

### 3.2 Changes in forest structure from 2012 to 2015

Wilcoxon paired sample test discovered that tree heights for all community forests varied significantly between 2012 and 2015. Except for APA‐KK, average tree heights increased slightly from 2012 to 2015 as in APA‐RC. Except for APA‐RC, the average crown diameters varied significantly between 2012 and 2015 and an increase in crown diameter was seen in all cases. The average values of these parameters (along with their standard deviations) have been provided in Table 2:

**Table 2:** Variation (Average ± *SD* **)** in LiDAR‐Derived Forest Structure Parameters (2012–2015)

	Average Height ± *SD* in 2012 (m)	Average Height ± *SD* in 2015 (m)	Average Crown Diameter ± *SD* in 2012 (m)	Average Crown Diameter ± *SD* in 2015 (m)	Gap Fraction % in 2012	Gap Fraction % in 2015	AGB (Mg/ha) in 2012	AGB (Mg/ha) in 2015
**APA‐KK**	13.76 ± 8.7	12.95 ± 8	6.4 ± 3.9	7 ± 4.3	48.6	26.6	100.46	274.38
**APA‐NT**	8.4 ± 4.7	8.7 ± 4.6	6.3 ± 4.16	6.6 ± 4.5	53.6	41	75.4	148.2
**APA‐RC**	12.7 ± 7	12.4 ± 6.7	6.5 ± 4	6.5 ± 4.1	40.2	36.7	154	180.7
**APA‐TD**	13.9 ± 7	14.6 ± 6.2	6.6 ± 3.9	6.7 ± 4	26.2	23	278.6	314
**CPA‐KK**	19.2 ± 8	19.3 ± 8.0	6.5 ± 3.7	6.6 ± 3.7	23.9	24.82	302.8	293.6
**CPA‐AT**	21.5 ± 6	21.8 ± 5.9	6.2 ± 3.22	6.4 ± 3.22	6.9	8.4	536.4	512.5

In addition to tree heights and crown areas, there was a statistically significant difference in the percentage gap fraction from 2012 to 2015 for all the community forests except APA‐TD. The gap fractions show signs of recovery in all the APAs except the CPAs and have increased slightly in the CPAs. Some of the AGB values decline in community forests while others increase (i.e., except for CPA‐KK and CPA‐AT where the AGB value declined by 3% and 4.4%, respectively, the AGB values of all the community forests increased significantly from 2012 to 2015).

In addition to the analysis of the variation in LiDAR‐derived forest canopy structure, ALOS PALSAR and Landsat data were also used to derive measures of forest degradation (RFDI) and green vegetation concentration (NDVI) across the community forests in the two time periods (Table 3).

**Table 3:** Variation in forest greenness and degradation (2012–2015)

	NDVI 2011 (%)	NDVI 2015 (%)	RFDI 2010 (%)	RFDI 2015 (%)
APA‐KK	54	70	58	57.7
APA‐NT	53	62	61.7	60
APA‐RC	55	65	56.7	57.1
APA‐TD	59	71	56.4	56.3
CPA‐KK	58	71.1	54	56.2
CPA‐AT	62	79	49	51

Notes

NVDI stands for Normalized Vegetation Index, APA stands for Archeological‐Protected Area which is classified into four such as Khlah Khmum (KK), Neak Ta (NT), Rong Chen (RC), and Thma Dap (TD), and CPA stands for Community‐Protected Area with its two classifications such as Khlah Khmum (KK) and Anlong Thom (AT).

NDVI was significantly different for all the community‐protected areas between 2012 and 2015, and it increased by 29.6%, 17%, 18.2%, 20.3%, 22.6%, and 24.1% for APA‐KK, APA‐NT, APA‐RC, APA‐TD, CPA‐KK, and CPA‐AT, respectively, indicating an increase in greenness of all community forests. RFDI was significantly different between 2012 and 2015 for APA‐NT, APA‐RC, CPA‐AT, and CPA‐KK. For both CPA‐AT and CPA‐KK, the RFDI increased by 4.17% and 3.7%, respectively, indicating a slight increase in forest degradation. RFDI of APA‐NT decreased by 3.2%, indicating a decline in forest degradation.

### 3.3 Impacts of selective logging

**Figure 6:**
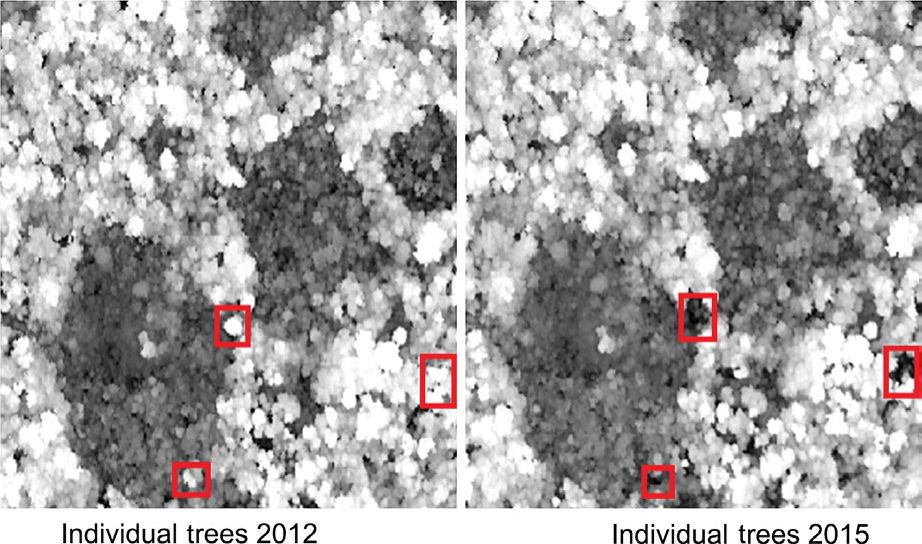
Individual tree difference in forest canopy of a community forest from 2012 to 2015

Visual inspection of the LiDAR‐derived CHM shows that individual canopy trees that were present in 2012 are no longer present in 2015 in the differently selected community forests (Figure 6):

Loss of individual trees in different community forests took place across the different tree height classes. CPA‐AT lost 3.46% trees with heights less than 10m. APA‐KK lost 4.5% trees in the height category 20–30 m and 3.15% trees in the height category 30–40 m. APA‐TD lost 5.12% and 5.7% of trees in categories 20–30 m and 30–40 m, respectively. The RDM values declined for all the community forests from 2012 to 2015 (Table 4).

**Table 4:** RDM values of community forests from 2012 to 2015

	RDM 2012 (%)	RDM 2015 (%)
**APA‐KK**	95	90
**APA‐NT**	97	87
**APA‐RC**	96	82
**APA‐TD**	96	90
**CPA‐KK**	96	48
**CPA‐AT**	95	49

The decline in RDM was 5.2%, 10.3%, 14.5%, 6.3%, 50%, and 48.4% in APA‐KK, APA‐NT, APA‐RC, CPA‐KK, and CPA‐AT, respectively. An analysis of road density obtained by digitizing Google Earth maps from 2003 to 2014 revealed that the community forests can be accessed via logging trails (see Supplementary Material 1).

## 4 DISCUSSION

### 4.1 Changes in forest cover and structure of the community forests

Analysis of the Landsat‐based binary forest–nonforest map revealed that all the community forests have much higher level persistence of forest cover persistence (72%–99%) compared to the wider landscape, where more than 20% of forest cover was lost in these three years. However, binary forest cover maps are unable to distinguish between different forest types, including plantation monocultures (Tropek et al., 2014). The 3 class Landsat‐LiDAR map also indicates a forest cover decline of 20% in the time period. These findings are consistent with the research done by Davies, Murphy, and Bruce (2016) in PKNP, which indicated an increase in the proportion of PKNP undergoing forest decline.

Unlike the binary forest cover class produced by ClaSLite, the 3 class LiDAR‐Landsat map helped spatially map forest areas converted to cashew plantations and indicated limited encroachment of these plantations into community forests. We note that the area under cashew plantations increased by 15% from 7.4 to 13.3 km^2^. Field research indicates that one of the community forests, APA‐NT, has high levels of cashew plantations, which had mainly been established before the APA was formally designated. Hence the cashew plantations observed within the community forest predate the period of analysis.

All community forests have also seen a significant increase in NDVI values which may be attributed to an increase in forest cover (Song, Huang, Sexton, Channan, & Townshend, 2014). NDVI is a robust indicator for mapping forest degradation, regeneration, and successional patterns and has been used for quantifying these in the tropical forests of Mexico and Congo (Hartter, Lucas, Gaughan, & Aranda, 2008; Njomo, 2008). The forest cover gain in the different community forests ranges from 0.2% to 24.6%. However, NDVI increases do not always correspond to forest cover increase which is why RFDI, a measure of forest degradation change was computed as well. It is remarked that in cases where an increase in NDVI corresponds to a decrease in RFDI (this being an indicator of decreasing forest degradation), it may be argued that forest regeneration may be underway. A combination of RFDI and Landsat‐based greenness measures was previously used to quantify the varying levels of degradation in a human‐modified tropical forest ecosystem in Lao PDR (Singh et al., 2017).

The analysis of LiDAR‐derived canopy heights and crown areas indicates that in all cases except one, these have increased in all the community forests. The canopy gap fractions have declined for all the APAs (but increased slightly for the CPAs).

While the AGB of the APAs has increased considerably from 2012 to 2015, it has undergone a slight decline in the CPAs (which have also had a slight increase in the canopy gap fractions). Previous research has established that regenerating forests can accumulate a large amount of carbon (Lasco, Visco, & Pulhin, 2001; Mukul et al., 2016). Forest regeneration and associated increases in forest cover facilitate the rapid increase in carbon stocks (Lohbeck, 2016). The decline in canopy gap fractions (and consequently an increase in canopy cover) is a sign of regeneration in tropical forests (Espirito‐Santo et al., 2006; Filer, Keenan, Allen, & Mcalpine, 2009). This is reflected in the AGB changes of the different community forests as well. Moreover, analysis of the ALOS‐derived forest degradation metric (RFDI) indicated that forest degradation has increased slightly in the CPAs and decreased in the APAs. It may be inferred that the increase in forest degradation (measured by RFDI) has had a detrimental effect on the canopy cover/gap fraction of the CPAs which in turn led to a decline in AGB stocks. Findings by Pfeifer et al. indicate that canopy cover (and AGB) declines as we move from virtually intact forests to logged forests to oil palm plantations (Pfeifer et al., 2016).

RFDI was previously used by Mitchard et al. (2012) to help distinguish between the different forest classes (Mitchard et al., 2011) and mapping the temporal variation in forest degradation (Joshi et al., 2015) in African forests. RFDI was also used for mapping forest degradation in the different forest types of human‐modified ecosystems of Laos (Singh et al., 2017). While developing formal relations between AGB and RFDI is not the focus of this research, the findings suggest that RFDI mapping can be undertaken as a way of identifying areas that have undergone high levels of degradation and are susceptible to losing their AGB stocks. This can be especially beneficial for monitoring degradation (and its impacts) in areas where LiDAR and other high‐resolution data sources are not available for fine‐scale AGB mapping.

Forest degradation is a spatially diverse phenomenon which unlike deforestation can also occur in forest ecosystems that have high or even near‐intact canopy coverage (Joshi et al., 2015). Our research backs up these findings; even though the community forests have retained >70% forest cover and see an increase in NDVI, the CPAs continue to suffer from small levels of degradation which is reflected in the increase in canopy gap fraction and decline in AGB stocks.

Selective logging for valuable tree species is a leading cause of forest degradation in the countries of Southeast Asia, including Cambodia (Miettinen, Stibig, & Achard, 2014). This has an adverse impact on forest structure parameters such as stand‐scale tree heights, AGB, gap fractions, and species composition (Asner et al., 2004; Gatti et al., 2015; Osazuwa‐Peters, Chapman, & Zanne, 2015; Rutishauser, Hérault, Petronelli, & Sist, 2016). However, selectively logged forests have been known to recover a substantial proportion of their AGB stocks within a few decades, although the impact still persists in the tree species composition and distribution after several decades (Gourlet‐Fleury et al., 2013; Ngo et al., 2013; Osazuwa‐Peters et al., 2015). A research of 175 1‐ha plots indicated that large dominant tree species contribute significantly to AGB stocks; 1.5% of the dominant species accounted for 50% of the AGB stocks in Africa (Bastin et al., 2015). However, since details of the individual tree species composition of the community forests are not known, it is not possible to measure how the removal of specific tree species influences AGB stocks in these forests. However, identifying and monitoring selective logging and its impacts can help to facilitate improved conservation management including the tracking of large trees in the target areas.

### 4.2 Monitoring selective logging

An examination of the prominent roads/logging trails presents within the study area indicates that all the community forests within the study area can be accessed using them and that many of the community forests are located in or near areas of high road density (see Supporting information Figure S1). It should be stressed that about two‐thirds of over 4,500 people that are living on the plateau across 10 villages are farmers who practice slash and burn clearing and cashew nut cultivation. When interviewed, park rangers stated that ongoing resource extraction from the community forests remains an ongoing concern. These interviews also confirmed the ongoing problem of selective luxury hardwood removal in PKNP, and that a pervasive network of roads/logging trails contributes substantially to that problem.

Interviewees also indicated that individual tree removal was being carried out on an ad hoc, opportunistic basis rather than a planned, systematic program of timber extraction. Future research will benefit from a detailed analysis of both the temporal changes in road density and its impact on individual tree removal and forest cover loss.

An analysis of relative density measure (RDM) also revealed that all community forests had faced an increase in understory damage caused by skidding and haulage, which are the hallmarks of selective logging for specific tree species (the RDM had decreased for all the community forests; Andersen et al., 2014; d'Oliveira, Reutebuch, McGaughey, & Andersen, 2012). However, the decline in RDM was especially large for the CPAs. This is arguably linked to an increase in forest degradation in these from 2012 to 2015 (measures using RFDI) and a consequent increase in the canopy gap fraction and decline in the AGB stocks. The RDM metric was previously used by Andersen et al. to quantify the increase in the area impacted by the hallmarks of selective logging‐ skidding trails, haulage, and roads from 2010 to 2011 in the Brazilian Amazon (Andersen et al., 2014). It was also discovered that areas impacted by these activities related to selective logging had a higher rate of AGB loss as compared to the nonimpacted areas (Andersen et al., 2014). This research also establishes that areas with a steep decline in RDM values lost AGB from 2012 to 2015. Additionally, the findings would indicate that the impact of selective logging in terms of increased skidding and understory damage is reflected in the RFDI metric, which is essentially based on the radar‐measured changes in the forest canopy (Mitchard et al., 2011; Saatchi et al., 2007). ALOS data are sensitive to patterns of disturbance and regrowth and were used to characterize these patterns from 2007 to 2010 for the Greater Mekong countries, including Cambodia (Chheng, Mizoue, Khorn, Kao, & Sasaki, 2015).

A previous study carried out in the semi‐evergreen forests of Cambodia revealed that during selective logging, felling of larger trees caused severe damage to the surrounding forest (Chheng et al., 2015). Meta‐scale analysis by Martin et al. revealed different logging techniques influence tree damage, AGB storage, and tree species dynamics differently (Martin, Newton, Pfeifer, Khoo, & Bullock, 2015). While we do not seek to establish any causality between RDM and radar‐measured degradation, this research has demonstrated the utility of different sources in evaluating forest loss and degradation. We suggest that temporal monitoring of forest cover change, especially in areas impacted by selective logging will benefit from evaluating the impact of different logging regimes and methods of felling different sized trees on the overall forest canopy. Use of multiscale remote sensing techniques (together with field data) can help to quantify forest degradation and its impact on both biodiversity and carbon (Bustamante et al., 2016).

### 4.3 Can community forests deliver conservation outcomes?

Our study area covers only a small proportion of PKNP. However, analysis of the study area does suggest that community‐protected forests are an effective bulwark against large‐scale deforestation/slash‐burn clearance and plantation establishment. Indeed, within our study area, the community forests had higher rates of forest cover retention as compared to areas outside the community forests. Furthermore, higher levels of forest‐to‐cashew plantation conversions were observed outside the community forests than inside. While our study area is relatively small and the changes were examined at a relatively narrow temporal scale, on the basis of these findings, it is suggested that analysis at larger spatial scales and longer temporal scales could be undertaken to better help to understand the spatiotemporal dynamics of forest cover change. Specifically, future research proposes to scale up the analysis to the scale of the whole of PKNP (and include other Cambodian PAs) to conduct a detailed analysis of forest cover retention and biodiversity conservation benefits provided by different protection and management schemes, including community forests and strict nature reserves.

A meta‐scale study of the community forests in South and South‐East Asia revealed that community forests have had a positive impact on improving tree species biodiversity and forest biomass production (Ravindranath, Murali, & Sudha, 2006). Previous research has discovered that community forests established in consultation with local people had higher AGB storage, reduced canopy openness and lower anthropogenic disturbance in the district of Prey Long in Cambodia (Lambrick et al., 2014). Policy research further suggests including local rulemaking autonomy may aid national scale REDD+ programs (Hayes & Persha, 2010), and that programs managed by local organizations garner more support from local people (Clements et al., 2010). Virachey National Park, an IUCN category II park located in northeastern Cambodia, partially encompasses the ancestral home of an ethnic minority group. It was discovered that the resource tenure regimes developed locally had a positive impact on biodiversity and local livelihood outcomes (Baird & Dearden, 2003). However, a significant drawback of this research is that it has not mapped the different forest types, especially forests that have faced varying levels of degradation. While this literature along with the findings of this research do indicate the positive conservation outcomes that community forests can deliver, future conservation prioritization may benefit from a more formalized comparison of community forests and more stricter categories. Future research will focus on identifying the role of management regimes (of both community forests and other management schemes) in delivering conservation outcomes.

Furthermore, it is important to identify and separate out plantations with the higher level of accuracy. A remote sensing‐based mapping of cashew plantations has not been undertaken before. Our research has demonstrated how a combination of remote sensing‐based data within a machine learning framework can help to differentiate land use types and facilitate the monitoring the changes in cashew plantation. Practical conservation management on the ground will significantly benefit from detailed mapping of the different forest types in the study area using multiple RS data sources and other machine learning algorithms.

In spite of the ability of the community forests to retain forest cover and allow for the recovery of the canopy cover (and associated AGB increases), the problem of selective logging of luxury hardwoods still persists within them. The removal of large trees can have an adverse impact on the AGB stocks and trees with DBH>70cm have a considerable impact on the AGB stocks of the different tropical forests across the globe (Slik et al., 2013). Not much is known about the specific hardwood species being harvested (or their size variations) in the study area. Hence, it cannot be ascertained how much AGB loss can be attributed to the removal of specific tree species. The possibility of forest patches losing AGB stocks as a result of fragmentation and edge effects, in the long run, cannot be ignored (Pütz et al., 2014).

A detailed analysis of field‐collected forest mensuration data in the mixed species Dipterocarp forests of Vietnam revealed that allometric equations developed for one site may overestimate AGB in other regions such as Indonesia, and allometric equations can produce more robust AGB estimates (Huy et al., 2016). One of the major shortcomings of the research is the lack of site‐specific forest mensuration data and allometric equations. Hence, while we can establish that community forests still continue to suffer from opportunistic selective logging of individual trees, we cannot quantify the impact of this on AGB stock variation. While field‐based plots are a cornerstone of modelling forest structure properties (especially those related to AGB storage and changes), it has been suggested that shifting to landscape scale aerial imagery‐based estimates could help to overcome the inherent biases of field inventory data (Marvin et al., 2014). This research demonstrates how different sources of RS data can help to map and monitor the temporal patterns of forest change. Their impact on forest structure properties at a relatively small scale investigated using both regeneration and degradation at different spatial resolutions. Future research will benefit from expanding analyses to the full area of the PKNP. Furthermore, this research focused on mapping the spatial distribution of three different forest types. Forests in PKNP have undergone varying levels of degradation and cashew plantations have different ages. Future research will also benefit by explicitly mapping forests that have undergone varying levels of degradation and are in varying stages of regeneration.

## 5 CONCLUSIONS

The temporal patterns of forest cover change, degradation, and recovery in community forests located within an IUCN Category II park have been examined using remotely sensed data acquired at different spatial resolutions. An examination of Landsat‐derived forest cover revealed that the community forests had higher forest cover persistence and lower rates of forest cover loss compared to the overall study area. The role of community forests in facilitating forest cover retention and regeneration is well established. The analysis of high‐resolution aerial LiDAR data also confirms these findings and except for the two CPAs, the remaining community forests have seen a recovery in canopy gap fractions and AGB stock. The patterns of fine‐scale canopy gap recovery and AGB stock increase are also reflected in the medium‐resolution forest degradation index derived from ALOS PALSAR data (RFDI). The levels of degradation decreased in forests that saw an increase in AGB values. While community forests have been shown to facilitate prevent cashew plantation expansion, forest cover persistence and recovery from degradation, illegal selective logging of individual tree species were not entirely curtailed within their boundaries. Analysis of LiDAR data indicates that all community forests experienced an increase in understory damage as a result of increased skidding and haulage (a sign of selective tree removal). Community forests with the sharpest increase in understory damage underwent AGB losses. An examination of individual trees identified by LiDAR data for 2012 and 2015 indicated a slight decline in the number of trees for the different classes. On the basis of these findings, it may be recommended that community‐protected forests can produce robust conservation outcomes in terms of forest cover persistence but more interventions are needed to curb the illegal selective logging of valuable timber trees.

## CONFLICT OF INTEREST

The authors declare no conflict of interest.

## AUTHORS’ CONTRIBUTIONS

Minerva Singh: Analyzed the data and wrote the manuscript. Damian Evans: Preprocessed the LiDAR data and helped to edit the manuscript. JB Chevance: Helped with fieldwork, provide study area details and edited the manuscript. Helped with study design. Mr. Nick Wiggins : Collection of field data and study design. Ms. Leaksmy Kong: Collection of field data and study design. Mr. Sakhoeun Sakada : GIS assistance and field data help. Dr. Boun Suy Tan : Facilitated the field study and study area history

## DATA AVAILABILITY

Data availability statement: Raw aerial data were generated through the Cambodian Archaeological LiDAR Initiative. Derived data supporting the findings of this study are present in the manuscript itself. The corresponding author [M.S.] will provide additional clarifications on request. Relevant data available via Dryad (https://doi.org/10.5061/dryad.nt7kg5g).
