# Supplementary material for: Evaluating the ability of community‐protected forests in Cambodia to prevent deforestation and degradation using temporal remote sensing data
Source: Ecol Evol. 2018 Oct 1;8(20):10175–91. doi: 10.1002/ece3.4492 (PMC6206189; doi:10.1002/ece3.4492)
Supplement: Supplementary file 1 [file ECE3-8-10175-s001.docx]

**Supplementary Material 1**

The spatial distribution of road density (km/sq km) of the most visible roads/logging rails:


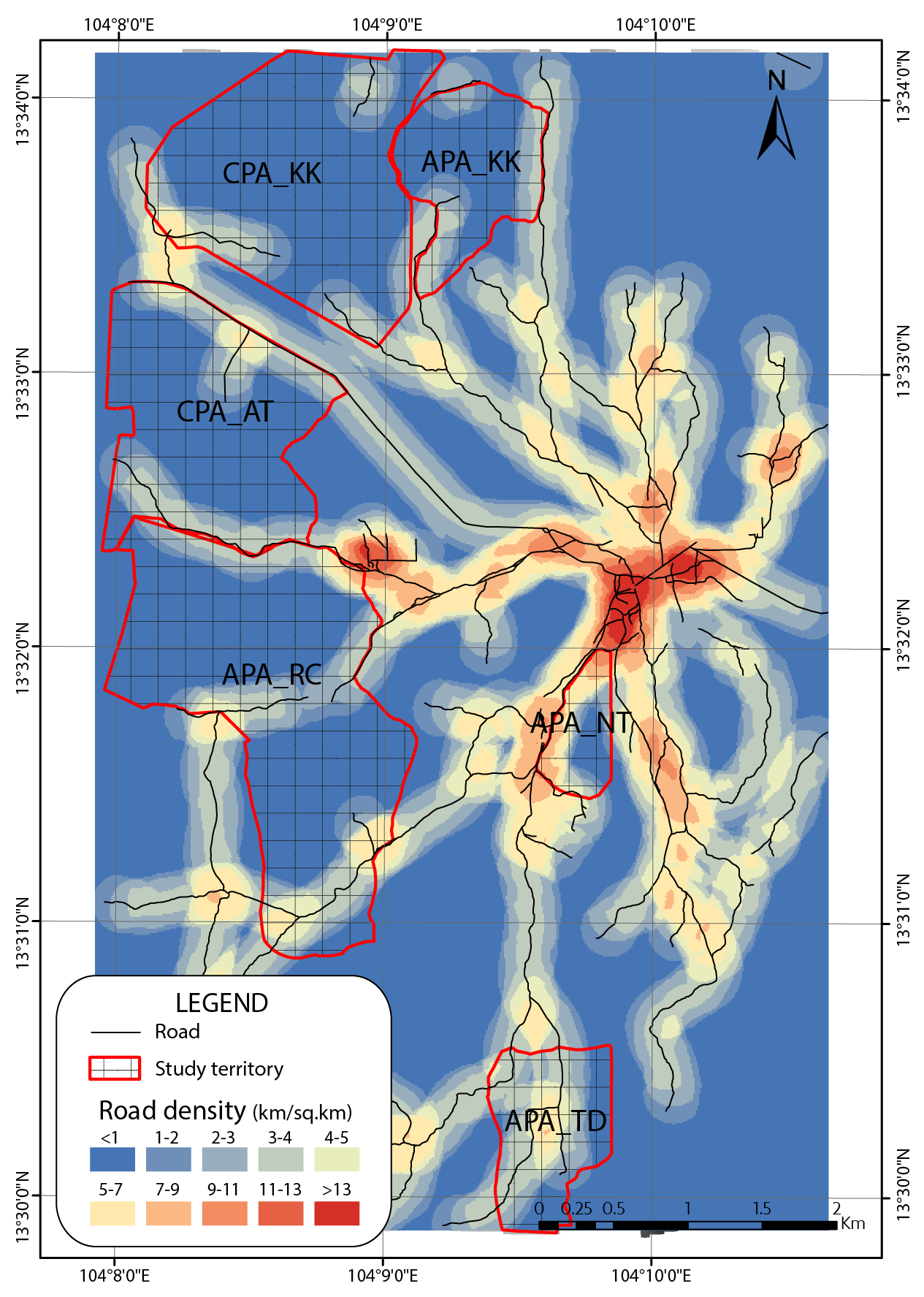


**Figure**

A visual inspection of the road density map (2013-2014) reveals that all the community forests can be accessed by logging trails/roads and these trails extend well into the forest. Some community forests such as APA-TD, APA-NT, APA-RC, CPA-AT and CPA-KK lie in the vicinity overlap with areas of medium to high road density.
